# Influenza Vaccine Effectiveness and Waning Effect in Hospitalized Older Adults. Valencia Region, Spain, 2018/2019 Season

**DOI:** 10.3390/ijerph18031129

**Published:** 2021-01-27

**Authors:** Ainara Mira-Iglesias, F. Xavier López-Labrador, Javier García-Rubio, Beatriz Mengual-Chuliá, Miguel Tortajada-Girbés, Joan Mollar-Maseres, Mario Carballido-Fernández, Germán Schwarz-Chavarri, Joan Puig-Barberà, Javier Díez-Domingo

**Affiliations:** 1 Fundación Para el Fomento de la Investigación Sanitaria y Biomédica de la Comunitat Valenciana (FISABIO-Public Health), 46020 Valencia, Spain; F.Xavier.Lopez@uv.es (F.X.L.-L.); javi.gr.81@gmail.com (J.G.-R.); mengual_bea@gva.es (B.M.-C.); jpuigb55@gmail.com (J.P.-B.); javier.diez@fisabio.es (J.D.-D.); 2 Consorcio de Investigación Biomédica de Epidemiología y Salud Pública (CIBERESP), Instituto de Salud Carlos III, 28029 Madrid, Spain; 3 Hospital Universitario Doctor Peset, 46017 Valencia, Spain; tortajadamig@gmail.com; 4 Hospital Universitario y Politécnico La Fe, 46026 Valencia, Spain; mollar_jua@gva.es; 5 Hospital General Universitario de Castellón, 12004 Castellón, Spain; carballido_mar@gva.es; 6 Departamento Medicina, Universidad CEU Cardenal Herrera, 12006 Castellón, Spain; 7 Hospital General Universitario de Alicante, 03010 Alicante, Spain; schwarz.ger@gmail.com; 8 Centro de Salud Pública de Castellón, 12003 Castellón, Spain

**Keywords:** influenza, vaccine, effectiveness, hospitalizations, waning, surveillance

## Abstract

Influenza vaccination is annually recommended for specific populations at risk, such as older adults. We estimated the 2018/2019 influenza vaccine effectiveness (IVE) overall, by influenza subtype, type of vaccine, and by time elapsed since vaccination among subjects 65 years old or over in a multicenter prospective study in the Valencia Hospital Surveillance Network for the Study of Influenza and other Respiratory Viruses (VAHNSI, Spain). Information about potential confounders was obtained from clinical registries and/or by interviewing patients and vaccination details were only ascertained by registries. A test-negative design was performed in order to estimate IVE. As a result, IVE was estimated at 46% (95% confidence interval (CI): (16%, 66%)), 41% (95% CI: (−34%, 74%)), and 45% (95% CI: (7%, 67%)) against overall influenza, A(H1N1)pdm09 and A(H3N2), respectively. An intra-seasonal not relevant waning effect was detected. The IVE for the adjuvanted vaccine in ≥75 years old was 45% (2%, 69%) and for the non-adjuvanted vaccine in 65–74 years old was 59% (−16%, 86%). Thus, our data revealed moderate vaccine effectiveness against influenza A(H3N2) and not significant against A(H1N1)pdm09. Significant protection was conferred by the adjuvanted vaccine to patients ≥75 years old. Moreover, an intra-seasonal not relevant waning effect was detected, and a not significant IVE decreasing trend was observed over time.

## 1. Introduction

Seasonal influenza is a common acute respiratory viral infection (ARVI) that can lead to serious complications and vaccination is key for its prevention [1]. Influenza vaccines are reformulated each season in response to changes in circulating strains and potential antigenic drift [1]. The World Health Organization (WHO) is the body responsible for recommending the vaccine composition for both hemispheres [2].

According to the data collected by the European Centre for Disease Prevention and Control (ECDC) across the WHO European Region, 99% of circulating influenza viruses in the 2018/2019 season were type A, with A(H1N1)pdm09 (57%) prevailing over A(H3N2) (43%), and 1% were type B. Almost all (1882/1885) genetically characterized A(H1N1)pdm09 viruses were subclade 6B.1, represented by the vaccine virus A/Michigan/45/2015. Most of the A(H3N2) circulating viruses in Europe belonged to subgroup 3C.2a1b (66%) represented by A/Alsace/1746/2018 and to clade 3C.3a (25%) represented by A/England/538/2018, both discordant with the egg-propagated A/Singapore/INFIMH-16-0019/2016 vaccine virus strain as The Francis Crick Institute, WHO Collaborating Centre, reported [3,4]. Only 3% of the 2163 specimens of the characterized A(H3N2) viruses were subclade 3C.2a1, represented in the influenza vaccine by the virus A/Singapore/INFIMH-16-0019/2016 [2,3].

Interim results from different studies across Europe for the 2018/2019 season showed a low vaccine effectiveness in hospitalized subjects aged 65 or more years old for both influenza A subtypes, A(H1N1)pdm09 and A(H3N2) [5]. Results from the USA in the same age range population were similar, but with moderate effectiveness in admissions with A(H1N1)pdm09 and showing no vaccine effectiveness in admissions with A(H3N2) [6].

In Spain, the Influenza Surveillance System reported that the 2018/2019 influenza season was characterized by the circulation of influenza A and very few cases of influenza B (less than 1%). The distribution by influenza A subtype was 45% A(H1N1)pdm09 and 55% A(H3N2). The peak of the season was reached in the week 2019-04, three weeks later than the previous two seasons [7].

The declining of effectiveness, also known as waning immunity, is considered a major concern around influenza vaccines. Our group, among other authors, have shown that the protection conferred by the influenza vaccine may wane [8,9], although the rate of this decline remains unclear. Previous vaccinations can also impair the protection of the influenza vaccine [10,11], especially for A(H3N2) [12].

In this study, we report the 2018/2019 end-of-season influenza vaccine effectiveness (IVE) in hospitalized patients 65 years old or over from the Valencia Region of Spain. Estimates are provided by influenza subtype and type of vaccine. We also explored the presence of a declining in the vaccine effectiveness over time.

## 2. Materials and Methods

### 2.1. Study Design

The study was carried out in 4 hospitals in the Valencia Region: Hospital General Universitario de Castellón (Castellón, Spain), Hospital Universitario y Politécnico La Fe (Valencia, Spain), Hospital Universitario Doctor Peset (Valencia, Spain) and Hospital General Universitario de Alicante (Alicante, Spain). Those hospitals provided healthcare to 1,118,732 (22%) inhabitants of the Valencia Region. The study encompassed the whole year although we restricted the analysis to the influenza season, defined by the time period between the first of two consecutive weeks with 2 or more influenza cases and the previous week to the first of two consecutive weeks with no influenza cases. The study methodologies have been described in previous publications [13]. Full-time dedicated nurses screened consecutive hospitalized patients discharged from the Emergency Department with complains possibly related to influenza (Supplement Table S1). Patients were included in the study if they were resident in the catchment area of one of the participating hospitals, non-institutionalized and not discharged from a previous admission in the last 30 days. Patients had to meet the ECDC influenza-like illness (ILI) case definition [14], defined as the presence of, at least, one systemic symptom (fever or feverishness, malaise, myalgia, or headache) and, at least, one respiratory symptom (shortness of breath, sore throat, or cough); onset of symptoms within seven days prior to admission and, finally, patients had to be in hospital between eight and 48 h to be approached and enrolled, after written informed consent.

### 2.2. Vaccine Information System

The Valencia Region Vaccine Information System (VRVIS) is a population-based registry that records all information related to vaccination. We can link this registry to sociodemographic information, Primary Care visits and hospitalizations through a unique personal identification number.

The VRVIS covers both public and private healthcare facilities. The sensitivity and specificity of the VRVIS were estimated previously for the influenza vaccine as 90% and 99% [13]. We obtained from the registry the influenza vaccine administration date, brand, batch, and manufacturer. We considered as immunized patients those who received the current season influenza vaccine at least 15 days before their symptoms’ onset. Not immunized vaccinated individuals were excluded from the analysis. Two different trivalent inactivated vaccines (TIV) were administered free of charge in the Valencia Region: Influvac^®^ (Mylan IRE Healthcare Limited, Dublin, Ireland) and Chiromas^®^ (Seqirus, S.R.L., San Martino, Italy), the second one included in its composition the adjuvant MF59C.1 [15,16]. Influvac^®^ was recommended for individuals 65 to 74 years old and Chiromas^®^ for individuals 75 years old and above and for institutionalized patients 65 years old or more [17], but only not institutionalized patients were included in our study as we considered institutionalization as an exclusion criteria. Data on previous influenza vaccinations was also obtained from the VRVIS.

### 2.3. Laboratory Procedures

Two different swabs, nasopharyngeal and pharyngeal (FLOQSwabs, Copan, Brescia, Italy), were obtained within the first 8–48 h of admission from all patients fulfilling the inclusion criteria. Both swabs were combined in one tube of viral transport media (Copan, Italy) and frozen until shipped refrigerated to a centralized Virology laboratory at FISABIO-Public Health. One third of the viral transport media volume was extracted for total nucleic acids using an automated silica-based method (Nuclisens Easy-Mag, BioMérieux, Lyon, France). Extracted nucleic acids were tested for influenza viruses by multiplex real-time reverse transcription-polymerase chain reaction (RT-PCR), following WHO protocols [18] with the qScript XLT One-Step RT-qPCR ToughMix (Quanta BioSciences, Beverly, MA, USA) in a Lightcycler 480II apparatus (Roche Diagnostics, Barcelona, Spain). First, a real-time RT-PCR screening assay was performed to detect and differentiate influenza A and B viruses using different primers and probes for the matrix protein [19]. Thereafter, two different real-time RT-PCR typing assays were performed to determine the viral subtype/lineage of influenza A or B viruses on influenza-positive samples [20,21].

Molecular characterization of influenza A(H1N1)pdm09 and A(H3N2) viruses was performed by whole-genome sequencing (WGS). All isolates from hospitalized cases with enough viral load (Ct < 27) were systematically selected and a universal viral whole-genome amplification protocol [22] was used to amplify the 8 segments of the viral genome simultaneously. The amplified fragments were desalted and used for generation of Illumina indexed Libraries by using the Nextera XT kit (Illumina, San Diego, CA, USA); and sequenced in 96-sample batches in an Illumina NextSeq platform (2 × 150 Mid-output kit). We used an in-house automated bioinformatic pipeline implemented in R [23] for demultiplexing, quality control, sequence pairing, assembly, generation of consensus sequences and variant analysis for each sample and segment of the viral genome; which is based in the use of prinseq-lite [24], FLASH [25], and snippy (https://github.com/tseemann/snippy) [26].

### 2.4. Genetic Analysis of Influenza Virus

Genetic characterization of influenza A(H1N1)pdm09 and A(H3N2) viruses was performed by comparison of the obtained consensus sequences for the complete HA coding region from the clinical isolates with representative and reference HA sequences (Supplement Table S2) obtained from the Global Initiative on Sharing Influenza Data (GISAID) database (www.gisaid.org). An alignment of reference sequences with sample sequences was generated with the Clustal W algorithm integrated in the BioEdit software ver. 7.2.5 (https://archive.org/details/bioedit). Phylogenetic trees were inferred using Maximum-likelihood methods and the best-fitting nucleotide substitution model with RAxML, and branch reliability was evaluated by approximate likelihood-ratio tests [27].

### 2.5. Statistical Analysis

#### 2.5.1. IVE Analysis

We explored the differences between laboratory-confirmed influenza (LCI) and non-LCI hospitalized patients in terms of age, sex, presence of chronic conditions (including pulmonary and heart diseases), admissions in the previous 12 months, number of General Practitioner (GP) visits in the previous 3 months, smoking habits, socioeconomic class according to occupation [28], obesity, days from symptoms onset to swabbing, previous influenza vaccinations, and current influenza vaccine immunization status. We used a Chi-squared test, a Fisher exact test or a non-parametric median test, as appropriate. All probabilities were two-tailed and *p*-values under 0.05 were considered statistically significant. An analogous descriptive analysis was performed comparing vaccinated and unvaccinated individuals. IVE was estimated following the test-negative design [29]. Cases were LCI admitted patients and controls were non-LCI admitted patients. The adjusted odds ratio (aOR) of being vaccinated between cases and controls was estimated through a logistic regression model including confounders identified for their biologic plausibility, for the “change-in-estimate” approach and following the parsimony principle [30,31]. We included age as a continuous covariate, sex, number of comorbidities (0, 1, 2, ≥3), presence of pulmonary and heart diseases, number of GP consultations in the last 3 months (0, 1, ≥2 visits), Barthel Index (Severe dependency 0–30, moderate dependency 35–65, mild dependency 70–90 and minimal dependency 95–100), epidemiological week at admission (categorical), previously vaccinated (considering prior 2 seasons) and hospital (categorical). The IVE was calculated as (1 − aOR) × 100% and the 95% confidence interval (CI) was also provided. Analogously, we repeated the analysis by vaccine type and for the different circulating viruses’ subtypes. In the subtype-specific IVE estimates, patients infected with other influenza subtypes were excluded as well as patients vaccinated with other influenza vaccine in the vaccine-specific analysis.

#### 2.5.2. Waning Effect Analysis

We explored the presence of an intra-seasonal waning of the vaccine effectiveness by using the number of elapsed days between vaccination and symptoms’ onset. We defined tertiles for that variable and we compared those categories with the reference category of no vaccination. For the waning effect analysis, we dropped out those patients who were not immunized when the influenza season started, in other words, we deleted patients not immunized at the onset of symptoms of the first detected influenza positive.

We proceeded as in the IVE analysis, adjusting a multivariate logistic regression by all potential confounders. The IVE for each time interval was calculated as (1 − aOR) × 100% and the 95% CI was also provided.

#### 2.5.3. Sensitivity Analysis

To better understand the impact of previous vaccination on current IVE we performed all the analysis restricting the sample size to those patients who were previously vaccinated in any of the prior two seasons.

All statistical analyses were carried out in Stata version 14 (StataCorp, College Station, TX, USA) and R (Vienna, Austria).

## 3. Results

### 3.1. Study Subjects and Influenza Season

**Figure 1:**
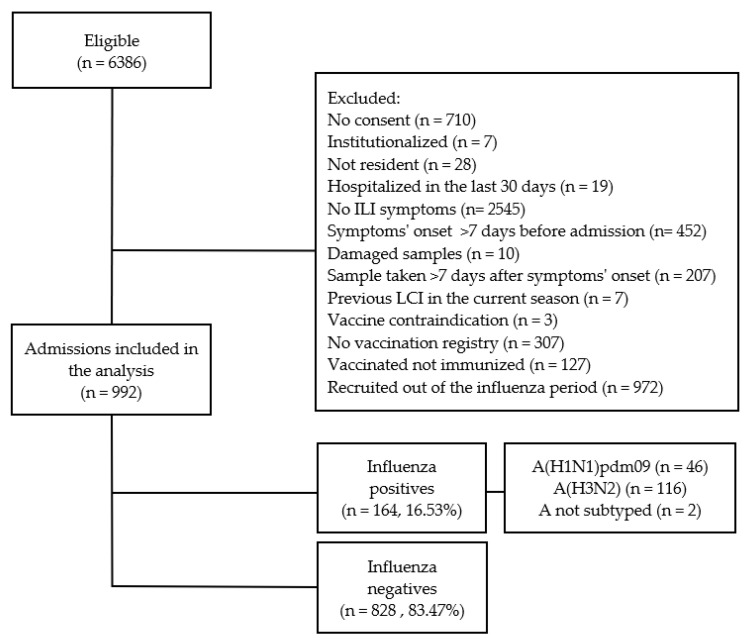
Selection process and influenza status of patients ≥65 years old for the influenza vaccine effectiveness (IVE) study. Results from the Valencia Hospital Network for the Study of Influenza (VAHNSI), Spain, in the 2018/2019 influenza season.

From 6836 eligible patients aged ≥65 years old, we included 992 patients in the IVE analysis after applying the exclusion criteria (Figure 1).

**Figure 2:**
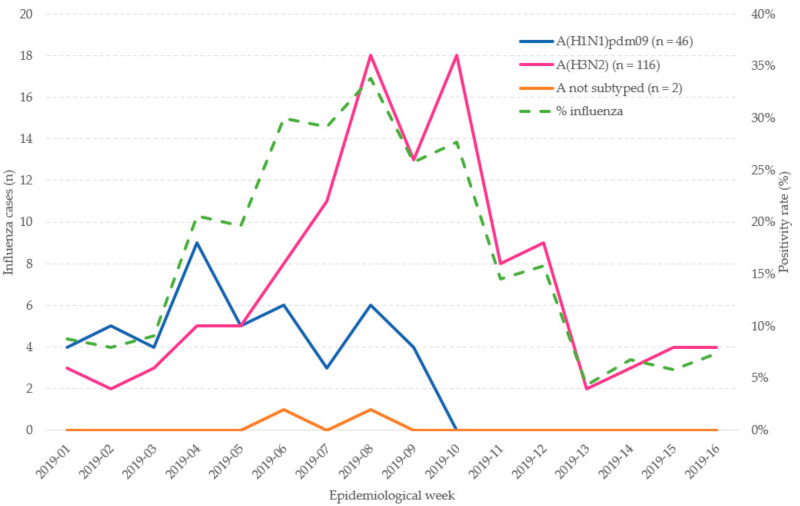
Admissions with laboratory-confirmed influenza by epidemiological week in patients ≥65 years old. Influenza positivity percentage by epidemiological week. Valencia Hospital Network for the Study of Influenza (VAHNSI), Spain, in the 2018/2019 influenza season.

The influenza period spanned from week 2019-01 to 2019-16. The first influenza wave was A(H1N1)pdm09, from 2019-01 to 2019-09, and the second and wider wave, from 2019-03 to 2019-16 was A(H3N2). The A(H1N1)pdm09 peaked in week 2019-04 and A(H3N2) peaked in 2019-08 and 2019-10 (Figure 2).

### 3.2. Influenza Positives vs. Influenza Negatives

From the 992 included patients, 164 (16.53%) were LCI: 46 (28.05%) A(H1N1)pdm09, 116 (70.73%) A(H3N2), and 2 A not subtyped (Figure 1). Influenza-positive patients were younger than negatives, median age 79 years old vs. 82 (Table 1).

Those patients resulting negative for influenza visited the GP more frequently than influenza positives (90.46% vs. 80.49%, *p*-value < 0.001). No differences between LCI and non-LCI were detected in terms of sex, presence of chronic conditions, pulmonary or heart diseases, admissions in the previous 12 months, smoking habits, socioeconomic class according to occupation, obesity, days from onset to swabbing, functional impairment status based on the Barthel Index and previous vaccination status. Positives and negatives for influenza differed in their vaccination status: 58.54% of positives were vaccinated vs. 68.36% of negatives (*p*-value = 0.015, Table 1).

### 3.3. Vaccinated vs. Unvaccinated Individuals

From the 992 included patients, 662 (66.73%) were vaccinated. Vaccinated individuals presented more chronic conditions than not vaccinated (96.68% vs. 92.42%, *p*-value = 0.011). Patients with pulmonary or heart diseases had higher vaccination coverage (71.93% vs. 63.68% among those with and without pulmonary diseases and 69.72% vs. 62.15% among those with and without heart diseases, data not shown). Males were more commonly vaccinated than females (70.89% vs. 62.42%, *p*-value = 0.005, data not shown). No differences between vaccinated and unvaccinated individuals were found regarding age, admissions in the previous 12 months, GP visits in the previous 3 months, smoking habits, socioeconomic class, obesity, days from onset to swabbing and functional impairment status (Table 1). Most of the vaccinated individuals (91.84%) were previously vaccinated (*p*-value < 0.001, Table 1).

### 3.4. A(H1N1)pdm09 Phylogenetic and Mutational Analyses

**Figure 3:**
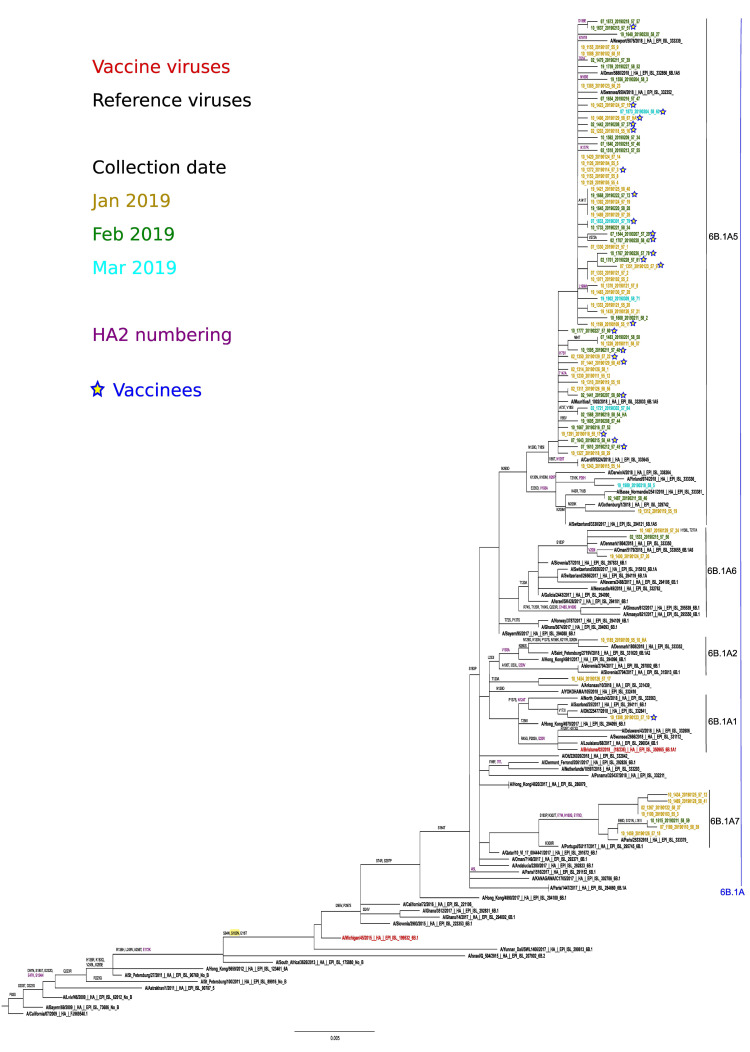
Phylogenetic analysis and mutational profiles for the A/H1N1pdm09 clades and sequenced isolates. Changes potentially gaining a glycosylation site are indicated in yellow. ML tree constructed using RAxML and the GTR-GAMMA substitution model.

The 35 A(H1N1)pdm09 viruses sequenced belonged to the A/Michigan/45/2015 (vaccine virus) clade 6B.1 which is defined by the amino acid substitutions S84N, S162N (potential glycosylation gain) and I216T (Figure 3).

Within clade 6B.1, viruses clustered into the 6B.1A subclade defined by additional substitutions S74R, S164T (alters glycosylation) and I295V. All viruses carried the substitution S183P, potentially linked to increased binding to α-2,6 sialic acid-linked receptors.

Within subclade 6B.1A several subclades showed different amino acid substitutions as compared to the vaccine virus. Three isolates clustered within A/Paris/2533/2018, subclade 6B1.A7 (characterized by K302T), and (HA2) I77M + N169S + E179D and one to A/Brisbane/02/2018 (2019-20 vaccine virus) subclade 6B1.A1. Finally, most of the isolates (*n* = 31) clustered to subclade 6B.1A5, characterized by N260D; with all of them close to A/Swansea/9504/2018 (clade 6B.1A5 (*), N129D + T185I). There was no evident grouping of isolates from the same collection month, or from vaccinated individuals within any of the observed subclades.

### 3.5. A(H3N2) Phylogenetic Analyses and Mutational Profiles

**Figure 4:**
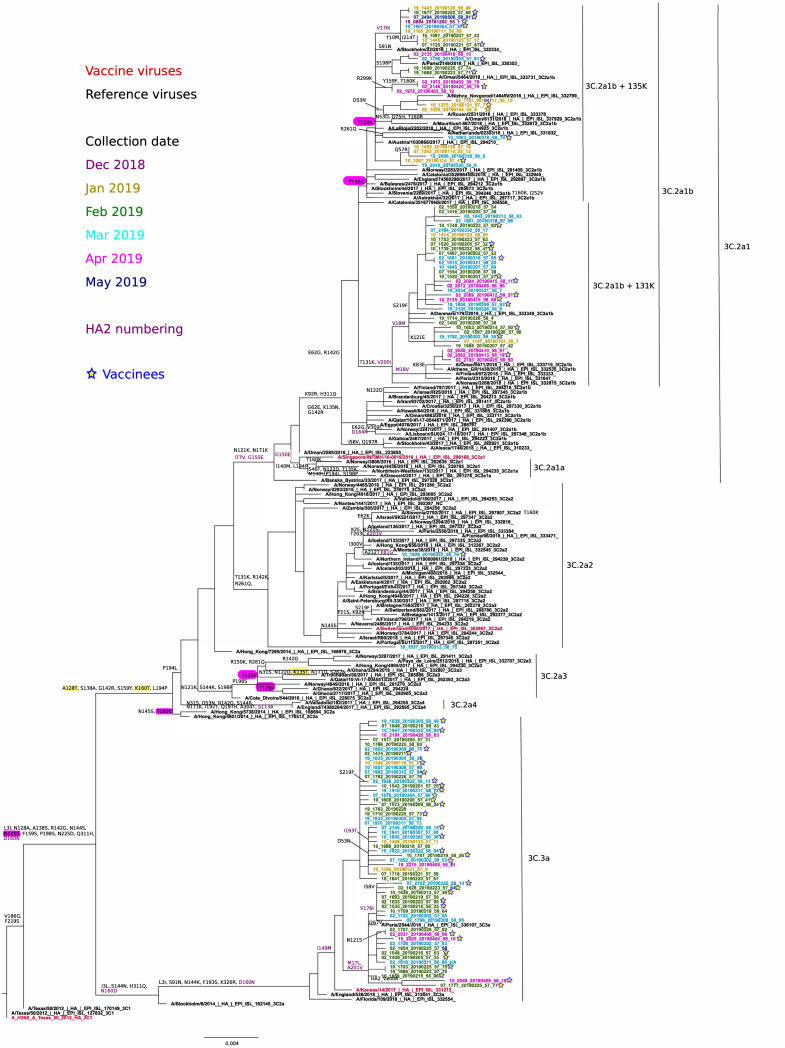
Phylogenetic analysis and mutational profiles for the A/H3N2pdm09 clades and sequenced isolates. Changes potentially gaining a glycosylation site are indicated in yellow, whereas those potentially losing a glycosylation site are indicated in red. ML tree constructed using RAxML (GTR-GAMMA substitution model).

The phylogenetic analysis of HA genes of circulating A(H3N2) viruses in the VAHNSI network and reference viruses indicated that all the 73 sequenced viruses fell within two clades: 3C.2a Hong Kong/4801/2014 (*n* = 35) and 3C.3a A/England/538/2018 (*n* = 38) (Figure 4).

There was no evident grouping of isolates from the same collection month, or from vaccinated individuals within any of the two clades. Only one 3C.2a isolate corresponded to subclade 3C.2a2 A/Switzerland/8060/2017. The 3C.2a2 viruses were defined by L3I, N128T (potential gain of glycosylation), T131K, R142K, N144S (potential loss of glycosylation), N145S, F159Y, K160T (potential gain of glycosylation at 158), P198S, F219S, R261Q, N225D, and (HA2)D160N.

Most 3C.2a viruses (*n* = 34) fell within subclade 3C.2a1, with no virus clustering in the same 3C.2a1 A/Singapore/INFIMH-16-0019/2016 (vaccine virus) branch; instead, all 3C.2a1 viruses further diversified on the closely-related A/Alsace/1746/2018 3C.2a1b subgroup. These viruses were characterized by substitutions E62G K92R, N121K/E, R142G, N171K, H311Q and (HA2)I77V, G155E, and G160E. Within this 3C.2a1b subgroup, two potentially differentiated subclades were observed: 3C.2a1b (*), (*n* = 19) with T131K + (HA2)V200I with or without HA2(V18M) and 3C.2a1b (**), (*n* = 15) with T135K (potential loss of glycosylation) together or not with T128A (potential loss of glycosylation). The 38 collected A(H3N2) 3C.3a viruses were in the A/England/538/2018 subgroup (which includes A/Kansas/14/2017, 2019-20 vaccine virus), and were defined by the substitutions L3I, S91N, T128A, A138S, most R142G, N144K (potential loss of glycosylation), most F193S, K326R and (HA2)D160N. We could distinguish two differentiating subgroups within 3C.3a isolates: 3C.3a (*), (*n* = 9) carried additional substitutions (HA2)M17L + A201V; and 3C.3a (**), (*n* = 6) carried additional substitutions (HA2)M17L + A201V + V176I.

### 3.6. Vaccine Effectiveness against Overall Influenza and for the Adjuvanted and Non-Adjuvanted Vaccines

The IVE for influenza vaccines overall against all influenza types in ≥65 years old hospitalized patients in the Valencia Region for the 2018/2019 season was 46.53% (95% CI: 16.49%, 65.77%). In particular, the IVE against all influenza types for the adjuvanted vaccine in those patients aged ≥75 was 44.81% (2.03%, 68.91%) and for the non-adjuvanted vaccine in patients 65–74 years old IVE was 59.50% (−16.33%, 85.90%) (Table 2).

### 3.7. Vaccine Effectiveness against A(H1N1)pdm09 and A(H3N2)

The IVE in ≥65 years old hospitalized patients in the Valencia Region for the 2018/2019 season was 40.65% (95% CI: −33.84%, 73.68%) against influenza A(H1N1)pdm09 and the IVE against A(H3N2) was 44.90% (7.40%, 67.21%) (Table 2).

### 3.8. Overall IVE Waning Effect

The waning effect was explored according to the number of days elapsed between vaccination and onset of symptoms dates. The following tertiles were defined: 15–82 days (first), 83–122 days (second), and 123–177 days (third). IVE was 54.17% (12.51%, 75.99%), 35.66% (−9.33%, 62.14%), and 49.83% (−2.77%, 75.51%) for patients in the first, second and third tertiles, respectively (Table 2).

### 3.9. Sensitivity Analysis

Restricting the analysis to those patients previously vaccinated (75% of total sample size) the overall IVE was 33.78% (−12.33%, 60.97%). For the study of the influenza effectiveness declining, we obtained that IVE was 18.72% (−78.93%, 63.08%), 26.71% (−34.09%, 59.94%), and 55.48% (−1.22%, 80.42%) for those patients whose elapsed days between vaccination and symptoms’ onset were between 15 and 82; 83 and 122; and 123 and 177 days, respectively. IVE against A(H1N1)pdm09 was −26.70% (−273.46%, 57.01%), and 40.10% (−8.50%, 66.93%) against A(H3N2). The IVE for the adjuvanted vaccine in those patients 75 years old or over was 40.72% (−17.24%, 70.03%) and for the non-adjuvanted vaccine in those adults 65–74 years old was 42.47% (−107.95%, 84.09%).

## 4. Discussion

In this work, we report the 2018/2019 IVE overall, by influenza subtype, vaccine type and by time elapsed since vaccination in hospitalized older adults in a multicenter prospective study in the Valencia Region of Spain.

We identified the A(H3N2) subtype as predominant, representing 71% of total isolations compared to 28% for A(H1N1)pdm09. Contrasting results were reported by the Canadian Sentinel Practitioner Surveillance Network that registered 88% of A(H1N1)pdm09 and only 6% of A(H3N2), and the United States IVE Network that detected 63% of A(H1N1)pdm09 and 22% of A(H3N2) in outpatients of all ages [32,33]. However, the distribution was practically the same for both A subtypes at the end of the season in the United States, with a latter predominance of influenza A(H3N2) viruses (49% of total positive isolations vs. 48% for A(H1N1)pdm09) [34]. In Europe, six studies combining primary care and hospitalizations reported a proportion of A(H1N1)pdm09 ranging between 58% and 80% [5], in agreement with the influenza circulation patterns reported by the WHO [35,36]. Nonetheless, data submitted by national influenza centers to The European Surveillance System (TESSy) revealed A(H3N2) predominance over A(H1N1)pdm09, 53% vs. 46% [3]. Published mid-term results from a sentinel network of general practitioners in Sicily (Italy) were also in line to ours: 62% A(H3N2) and 26% A(H1N1)pdm09 [37]. Therefore, temporal and geographical variations on circulating influenza viruses were identified during the 2018/2019 influenza season. Differences among study populations in terms of health-care setting, age, and presence of confounding factors could also be influencing that variability.

The WHO recommended composition of the TIV for the northern hemisphere included an A/Michigan/45/2015 (H1N1)pdm09-like virus, an A/Singapore/INFIMH-16-0019/2016 (H3N2)-like virus, and a B/Colorado/06/2017-like virus (B/Victoria lineage). In our study, 44% of the A(H3N2) viruses genetically characterized were similar to A/Alsace/1746/2018 (clade 3C.2a1b), and 55% of them to A/England/538/2018 (clade 3C.3a). The A(H3N2) vaccine component A/Singapore/INFIMH-16-0019/2016 is the clade 3C.2a1 representative, that replaced the clade 3C.2a A/Hong Kong/4801/2014 virus used in the previous 2016/17 and 2017/18 seasons. Despite genetic differences in the HA1 region, it is believed that 3C.2a and 3C.2a1 vaccine strains may be antigenically similar, but different to contemporary 3C.2a1b and 3C.3a viruses, with 3C.3a viruses showing greater antigenic differences [4,38]. Thus, some kind of cross-reactivity between the 3C.2a1 A/Singapore/INFIMH-16-0019/2016 vaccine virus and the 3C.2a1b viruses that circulated in 2018/19 may be possible. In addition, one can also hypothesize a potential beneficial effect of previous vaccination in 2016/17 and 2017/18 seasons because the A(H3N2) component in the vaccine was A/Hong Kong/4801/2014 (3C.2a), which may have rendered some cross-reactivity with the 3C.2a1b (but not with the 3C.3a) circulating viruses in 2018/19. To support this idea, it is interesting to note that clade-specific IVEs in other countries with co-circulation of 3C.2a1b and 3C.3a A(H3N2) viruses were clearly higher for 3C.2a/3C.2a1b clades (with positive values reaching even 58%) as compared to those for 3C.3a (mostly very low or negative) [38,39,40]. Unfortunately, sample size limitations precluded clade-specific IVE calculations in our study. In contrast, all the A(H1N1)pdm09 characterized viruses in our study belonged to clade 6B.1, which was antigenically similar to the A/Michigan/45/2015 strain included in the vaccine [7]. Several recently published studies reported similar results [3,34].

In terms of IVE results, for hospitalized patients 65 years old or over from the Valencia Region of Spain we estimated an IVE of 46%. Interestingly, differences were found across regions or countries. Vaccine effectiveness against overall influenza was 53% (40%, 64%) in England and 35% (17%, 49%) in Denmark in hospitalized older adults [5,41]. Lower effectiveness, 26% (1%, 45%), was found in another study in Northern Spain [42]. Despite of virological characterization of the viruses revealing some genetic differences with the vaccine strain, we found significant protection (45%) against influenza A(H3N2) as well as other study in England that reported an IVE of 39% (6%, 61%), were clade 3C.2a1 viruses were clearly predominant [41]. When analyzing clade-specific IVEs, other studies revealed indeed significant protection for clade 3C.2a1 viruses but negligible protection for 3c.3a viruses [38,40]. These observations emphasize the urgent need for large studies calculating IVEs considering the genetic characterization of circulating viruses and the antigenic differences between them and vaccine strains each year.

Conversely, we did not detect significant IVE against influenza A(H1N1)pdm09, probably due to low sample size when restricting the analysis, although our point estimate of 41% was close to the one reported in Denmark (37% (3%, 60%)) [5].

Two different vaccines were administered in the Valencia Region during the 2018/2019 influenza season. We estimated the overall IVE for the adjuvanted vaccine in patients 75 years old or over, the target group for that vaccine. We found significant protection (45%) as well as other published works in older adults population from the United Kingdom both in hospitalizations and primary care with an IVE of 54% (40%, 64%) and 62% (3%, 85%), respectively [39,41]. On the contrary, we did not find significant protection for the non-adjuvanted vaccine in adults 65–74 years old.

Most studies reporting IVE estimates did not consider previous vaccinations in the regression model [5,34,37,39,41], although that information is relevant and valuable. We had the opportunity to obtain vaccination histories of patients through population registries [13]. Other authors performed additional analyses by establishing different categories of vaccination status considering current and previous seasons, and taking as reference patients not vaccinated either in the current or prior seasons [42]. In our perspective, this approach generates groups which are “not comparable” but end up being compared, such as patients that always get vaccinated and patients that never get vaccinated, are being compared. We proposed the alternative of including prior vaccination in the IVE model as a covariate. We also explored the interaction term between current and prior vaccinations, but we did not obtain better results. As most of the older adult population was annually vaccinated, we carried out a sensitivity analysis to estimate the IVE only in patients previously vaccinated. However, due to sample size reduction we did not obtain significant results, although the point estimates for IVE against A(H3N2) and for the adjuvanted vaccine were similar to the ones obtained in the main analysis.

We found a considerable heterogeneity in the results of published studies on repeated vaccination, most possibly due to different populations, variability of vaccine composition and circulating virus in the considered seasons [12]. Older adults already vaccinated in the two previous seasons did not seem to be protected against influenza A(H1N1)pdm09 [43]. A negative impact of repeated vaccination on vaccine-induced protection was also found against influenza A(H3N2), although the authors remarked some limitations as the covered short time period, the small number of included studies, the low precision of the estimates, and the heterogeneity in terms of study design, population and setting, vaccination type and ascertainment and seasons [12]. Conversely, no negative effects of repeated seasonal vaccination were detected in Sweden when studying a large population-based cohort of hospitalized older adults, supporting the recommendation of being vaccinated annually [44]. Smith et al. proposed the antigenic distance hypothesis as an explanation for the effect of repeated vaccination [45], with positive and negative “interferences” of previous vaccination according to the match of previous and current vaccine strains with the circulating strain. Monto et al. suggested the term “antigenic interaction” instead to better describe the phenomenon proposed by Smith et al. [46]. The variability in the literature examining repeated vaccination reflects the necessity of developing longitudinal studies on vaccination history, exploring immunogenicity and long-term protection in different seasons and populations.

The presence of an intra-seasonal waning effect of the vaccine immunization was usually detected when exploring IVE against A(H3N2) [9,47,48]. We observed that trend against overall influenza during the 2018/2019 season, with overlapping confidence intervals. Some publications raised the concern that individuals vaccinated early in the season could become vulnerable to influenza infection when the virus reaches its maximum activity [49]. That fact could be due to different factors such as the loss of immunity or the evolution of the virus during the influenza season. However, statistical artefacts or depletion of susceptible bias cannot be ruled out [47,50]. Although some public health authorities have also implemented a later start of influenza vaccination programs (in comparison to previous seasons), some authors are highly reluctant to act on the existing evidence [47,51].

Our study was subject to different biases as it is usual in observational studies. We tried to avoid selection bias, including all patients fulfilling a well-established case definition. Vaccination status was ascertained by registries, influenza infection was laboratory-confirmed with a sensitive real-time RT-PCR assay, and only patients swabbed within 8–48 h of admission in hospital were included to avert misclassification bias. Unknown confounding can bias results in observational studies. We tried to control confounders by adjusting models by factors related to both vaccination status and influenza result. Nevertheless, residual confounding always remains as some confounder are not measurable. In some of the analysis, a larger sample size would allow us to provide more robust estimates.

## 5. Conclusions

Our data revealed a moderate IVE, although an intra-seasonal not relevant waning effectiveness trend was observed, in hospitalized patients 65 years old or over in the Valencia Region of Spain during the 2018/2019 season. IVE by subtype ranged between 41 and 45%, although protection was not significant against A(H1N1)pdm09. The effectiveness of the adjuvanted vaccine was also proved in patients 75 years old or over, which represent the target population for receiving that vaccine.

## Figures and Tables

**Table 1:** Characteristics of included admissions in the Valencia Hospital Network for the Study of Influenza (VAHNSI) in the 2018/2019 season.

Characteristics	Influenza Positives		Influenza Negatives		*p*-Value	Vaccinated Individuals		Unvaccinated Individuals		*p*-Value
	*n*	%	*n*	%		*n*	%	*n*	%	
Overall	164	100	828	100		662	100	330	100	
Age (median, (IQR))	79	(74–86)	82	(75–87)	0.029	81	(75–87)	82	(74–88)	0.576
Age group					0.171					0.782
65–74	49	29.88	194	23.43		158	23.87	85	25.76	
75–84	61	37.20	314	37.92		254	38.37	121	36.67	
≥85	54	32.93	320	38.65		250	37.76	124	37.58	
Sex					0.796					0.005
Male	85	51.83	420	50.72		358	54.08	147	44.55	
Female	79	48.17	408	49.28		304	45.92	183	55.45	
Chronic conditions					0.836					0.011
0	9	5.49	38	4.59		22	3.32	25	7.58	
1	39	23.78	183	22.10		143	21.60	79	23.94	
2	49	29.88	239	28.86		192	29.00	96	29.09	
≥3	67	40.85	368	44.44		305	46.07	130	39.39	
Pulmonary diseases (other than asthma)					0.767					0.008
No	105	64.02	520	62.80		398	60.12	227	68.79	
Yes	59	35.98	308	37.20		264	39.88	103	31.21	
Heart diseases					0.102					0.013
No	74	45.12	317	38.29		243	36.71	148	44.85	
Yes	90	54.88	511	61.71		419	63.29	182	55.15	
Admissions in the previous 12 months					0.107					0.480
No	111	67.68	505	60.99		406	61.33	210	63.64	
Yes	53	32.32	323	39.01		256	38.67	120	36.36	
GP visits in the previous 3 months					<0.001					0.543
0	32	19.51	79	9.54		71	10.73	40	12.12	
1	21	12.80	65	7.85		54	8.16	32	9.70	
≥2	111	67.68	684	82.61		537	81.12	258	78.18	
Smoking habits					0.837					0.165
Never smoker	95	57.93	498	60.14		383	57.85	210	63.64	
Ex-smoker	57	34.76	277	33.45		231	34.89	103	31.21	
Current smoker	12	7.32	53	6.40		48	7.25	17	5.15	
Socioeconomic class (occupation)					0.230					0.701
Professional	23	14.02	143	17.27		107	16.16	59	17.88	
Skilled	6	3.66	51	6.16		40	6.04	17	5.15	
Unskilled	135	82.32	634	76.57		515	77.79	254	76.97	
Obesity					0.764					0.098
No	117	71.34	581	70.17		477	72.05	221	66.97	
Yes	47	28.66	247	29.83		185	27.95	109	33.03	
Days from onset to swabbing					0.18					0.915
1–2	33	20.12	194	23.43		153	23.11	74	22.42	
3–4	82	50.00	349	42.15		289	43.66	142	43.03	
5–7	49	29.88	285	34.42		220	33.23	114	34.55	
Functional impairment status (Barthel Index)					0.117					0.595
Total-severe (0–30)	18	10.98	153	18.48		111	16.77	60	18.18	
Moderate (35–65)	17	10.37	76	9.18		66	9.97	27	8.18	
Mild (70–90)	31	18.90	128	15.46		101	15.26	58	17.58	
Minimal (95–100)	98	59.76	471	56.88		384	58.01	185	56.06	
Vaccination status					0.015					NA
No	68	41.46	262	31.64		NA	NA	NA	NA	
Yes	96	58.54	566	68.36		NA	NA	NA	NA	
Previous vaccinations (2 seasons)					0.742					<0.001
No	43	26.22	207	25.00		54	8.16	196	59.39	
Yes	121	73.78	621	75.00		608	91.84	134	40.61	
Positives for influenza					NA					
A(H1N1)pdm09	46	28.05	NA	NA		21	3.17	25	7.58	0.002
A(H3N2)	116	70.73	NA	NA		73	11.03	43	13.03	0.355
A not subtyped	2	1.22	NA	NA		2	0.30	0	0.00	0.318

IQR: interquartile range. GP: general practitioner. NA: not applicable.

**Table 2:** Influenza vaccine effectiveness by strain, vaccine type and time since vaccination in hospital admissions (65 years old or over). Results from the Valencia Hospital Network for the Study of Influenza (VAHNSI) in the 2018/2019 influenza season.

IVE Estimates	Cases				Controls				IVE (%)	95% CI
	Vaccinated		Unvaccinated		Vaccinated		Unvaccinated			
	N	%	N	%	N	%	N	%		
1. IVE against overall influenza ^a^	96	58.54	68	41.46	566	68.36	262	31.64	46.53	(16.49, 65.77)
2. IVE against A(H1N1)pdm09 ^a^	21	45.65	25	54.35	330	67.90	156	32.10	40.65	(−33.84, 73.68)
3. IVE against A(H3N2) ^a^	73	62.93	43	37.07	566	68.36	262	31.64	44.90	(7.40, 67.21)
4. MF59-adjuvanted (in ≥75 years old) IVE against overall influenza ^a^	44	50.57	43	49.43	261	56.37	202	43.63	44.81	(2.03, 68.91)
5. Non-adjuvanted (in 65–74 years old) IVE against overall influenza	20	46.51	23	53.49	83	63.36	48	36.64	59.50	(−16.33, 85.90)
6. IVE against overall influenza according to days between vaccination and symptoms’ onset dates ^b^:										
Not vaccinated	NA	NA	68	100	NA	NA	262	100	Ref.	
15–82 days	21	21.88	NA	NA	191	33.75	NA	NA	54.17	(12.51, 75.99)
83–122 days	55	57.29	NA	NA	167	29.51	NA	NA	35.66	(−9.33, 62.14)
123–177 days	19	19.79	NA	NA	196	34.63	NA	NA	49.83	(−2.77, 75.51)

IVE: influenza vaccine effectiveness; CI: confidence interval; NA: not applicable; Ref: reference category. Bold means significant IVE estimates. ^a^: row percentage; ^b^: column percentage; Models 1–4 and 6 were adjusted by age, sex, number of chronic conditions, pulmonary underlying disease, heart underlying disease, number of GP visits in the last 3 months, Barthel Index, epidemiological week at admission, previous vaccination and hospital. Model 5 was adjusted by epidemiological week at admission and previous vaccination. Controls numbers differed for A(H1N1)pdm09 due to circulation period restriction, for the MF59-adjuvanted vaccine due to target age groups restriction, and for the non-adjuvanted vaccine due to circulation period and target age groups restriction. For the waning effect analysis, vaccinated patients not immunized at the beginning of the influenza season were discarded.

## Data Availability

The data presented in this study are available on request from the corresponding author. The data are not publicly available due to privacy restrictions.

## Supplementary Materials

The following are available online at https://www.mdpi.com/1660-4601/18/3/1129/s1, Table S1: Admission diagnoses of the Valencia Hospital Network for the Study of Influenza (VAHNSI), Table S2: Sequences from GISAID’s EpiFlu™ Database (1) on which this research was based.
