# Supplementary material for: Influenza Vaccine Effectiveness and Waning Effect in Hospitalized Older Adults. Valencia Region, Spain, 2018/2019 Season
Source: Int J Environ Res Public Health. 2021 Jan 27;18(3):1129. doi: 10.3390/ijerph18031129 (PMC7908304; doi:10.3390/ijerph18031129)
Supplement: Supplementary file 1 [file ijerph-18-01129-s001.zip › Table S1.docx]

| **Eligibility diagnoses, symptoms and signs** | **ICD 10 Codes** |
| --- | --- |
| Acute respiratory infection | J00-J06, J20-J22, H66.90 |
| Acute myocardial infarction or acute coronary syndrome | I20-I25.9 |
| Asthma | J45.2-J45.22, J45.9-J45.998, J44-J44.9 |
| Heart failure | I50-I50.9, I51.4 |
| Pneumonia and influenza | J09-J18 |
| Chronic pulmonary obstructive disease | J40-J44.9 |
| Myalgia | M79.1 |
| Metabolic failure (diabetic coma, renal dysfunction, acid-base disturbances, alterations of the water balance) | E10.10, E10.11, E10.641, E10.65, E10.69, E10.9, E11.00, E11.01, E11.641, E11.65, E11.69, E11.9, E86.0, E86.1, E87.0, E87.1, E87.2, E87.3, E87.4, E87.5, E87.6, E87.70, E87.71, E87.79, N17.0, N17.1, N17.2, N17.8, N17.9, N18.1, N18.2, N18.3, N18.4, N18.5, N18.6, N18.9, N19 |
| Altered consciousness, convulsions, febrile convulsions | R40.0, R40.1, R40.20, R40.4, R56.00, R56.01 |
| Dyspnoea / respiratory abnormality / shortness of breath / respiratory symptoms | R06-R06.9 |
| Fever or fever of unknown origin or non-specified | R50, R50.9 |
| Cough | R05 |
| Apnoea | R06.81 |
| Sepsis, Systemic inflammatory response syndrome | A41.9, R65.10, R65.11, R65.20 |

**Table S1**: Admission diagnoses of the Valencia Hospital Network for the Study of Influenza (VAHNSI).
